# Real-Time Acoustic Scene Recognition for Elderly Daily Routines Using Edge-Based Deep Learning

**DOI:** 10.3390/s25061746

**Published:** 2025-03-12

**Authors:** Hongyu Yang, Rou Dong, Rong Guo, Yonglin Che, Xiaolong Xie, Jianke Yang, Jiajin Zhang

**Affiliations:** 1 College of Mechanical and Electrical Engineering, Yunnan Agricultural University, Kunming 650201, China; 2 Center for Sports Intelligence Innovation and Application, Yunnan Agricultural University, Kunming 650201, China; 3 College of Physical Education, Yunnan Agricultural University, Kunming 650201, China; 4 College of Big Data, Yunnan Agricultural University, Kunming 650201, China

**Keywords:** acoustic scene recognition, edge computing, deep learning, health monitoring, Internet of Things (IoT)

## Abstract

The demand for intelligent monitoring systems tailored to elderly living environments is rapidly increasing worldwide with population aging. Traditional acoustic scene monitoring systems that rely on cloud computing are limited by data transmission delays and privacy concerns. Hence, this study proposes an acoustic scene recognition system that integrates edge computing with deep learning to enable real-time monitoring of elderly individuals’ daily activities. The system consists of low-power edge devices equipped with multiple microphones, portable wearable components, and compact power modules, ensuring its seamless integration into the daily lives of the elderly. We developed four deep learning models—convolutional neural network, long short-term memory, bidirectional long short-term memory, and deep neural network—and used model quantization techniques to reduce the computational complexity and memory usage, thereby optimizing them to meet edge device constraints. The CNN model demonstrated superior performance compared to the other models, achieving 98.5% accuracy, an inference time of 2.4 ms, and low memory requirements (25.63 KB allocated for Flash and 5.15 KB for RAM). This architecture provides an efficient, reliable, and user-friendly solution for real-time acoustic scene monitoring in elderly care.

## 1. Introduction

Over the past few decades, many countries have experienced a significant increase in the proportion of elderly individuals, reflecting the global trend of population aging [1]. According to data from the World Health Organization, the global population aged 60 years and older is projected to reach approximately 2 billion by 2050 [2]. China’s elderly population aged 60 years and above had reached 212 million by the end of 2014, and is expected to reach 358 million by 2030, accounting for more than 25.3% of the total national population [3]. This presents severe challenges in enhancing the quality of life of the elderly and addressing their health and well-being. The monitoring of daily activities and habits of elderly people is crucial for assessing their overall health status, monitoring disease development, and providing personalized care services [4,5]. However, the provision of continuous private care has obvious effects on the independence and psychological status of the elderly, and it also imposes a financial burden on families and society.

Traditional monitoring methods, such as video surveillance and routine health check-ups, provide extensive coverage, but also present the challenges of privacy concerns device maintenance, and data processing [6,7]. Acoustic scene recognition (ASR), a promising alternative to address these challenges [8], can accurately detect and classify different acoustic scenes by analyzing ambient sound data in real time, thereby ensuring comprehensive monitoring of the surroundings of elderly individuals [9]. This approach enhances real-time safety monitoring while also delivering detailed scene insights, reducing the dependence on visual data and addressing privacy concerns more effectively [10,11].

With advances in artificial intelligence technology, machine and deep learning have been extensively applied in the field of ASR [12,13]. Traditional machine learning algorithms, such as support vector machine (SVM), random forest (RF), decision trees, and k-nearest neighbors (k-NN), have been utilized in this domain [14,15]. For instance, various feature extraction algorithms, such as Mel-frequency cepstral coefficient (MFCC), Gammatone Cepstral Coefficients (GTCCs), and Naive Bayes (NB), were compared with machine learning algorithms for acoustic event detection across five different acoustic environments [16]. The results indicated that most algorithm combinations performed well, with the GTCC and k-NN combination achieving the best performance. Lei and Mak [17] designed an energy-efficient sound event detector that utilized sound event partitioning (SEP) techniques and employed SVM for classification. Abidin et al. [18] proposed an acoustic scene classification method that combines local binary patterns (LBPs) with RF. They first applied the constant-Q transform (CQT) to audio signals by dividing the spectrum into multiple sub-bands to capture local spectral features. The LBP was then used to extract these time–frequency features, and RF was employed for classification, achieving a classification accuracy of 85.0%. Although these methods, which rely on manually extracted features to classify acoustic scenes, have demonstrated good performance in certain tasks, their ability to capture complex patterns and high-dimensional features in sound data remains limited.

Deep learning algorithms, particularly convolutional neural networks (CNNs), automated feature extraction, and end-to-end learning, enable networks to extract hierarchical features from raw sound data and capture local patterns and textures in audio signals. This significantly enhances the recognition accuracy [19,20]. Vivek et al. [21] designed a sound classification system using an automatic hearing aid switching algorithm, thereby enabling traditional hearing aids to adapt to varying sound environments. This system utilized features such as MFCC, Mel spectrogram, and chroma, and trained a CNN to classify five sound categories with high accuracy and efficiency while incurring low memory cost. Zhu et al. [22] proposed a deep CNN architecture for environmental sound classification that processes raw audio waveforms and employs a parallel CNN to capture features at different temporal scales. The architecture also incorporates direct connections between layers to improve information flow and mitigate the vanishing gradient problem. Basbug and Sert [23] integrated the spatial pyramid pooling (SPP) method within CNN convolutional layers to aggregate local features, using MFCC, Mel energy, and spectrograms as input features. The results showed that the CNN-SPP architecture with spectrogram features improved classification accuracy. Furthermore, Tripathi and Mishra [24] introduced a novel model for environmental sound classification, incorporating an attention mechanism that focuses on semantically relevant frames in spectrograms and learning spatiotemporal relationships within the signal. This study on the ESC-10 and DCASE 2019 Task-1(A) datasets demonstrated improvements of 11.50% and 19.50%, respectively, over baseline models. These discussions highlight that applying deep learning techniques can provide a theoretical foundation and technological support for ASR in elderly monitoring, thereby offering more precise safety surveillance.

Additionally, with the advancement of Internet of Things (IoT) technologies and improvement in the computational power and storage resources of edge devices, it is feasible to deploy deep learning models on these devices [25,26]. Acoustic scene changes can be detected efficiently by performing real-time inferences on edge devices, thereby providing more accurate and timely monitoring services. This approach reduces the dependency on cloud computing, thus lowering data transmission latency and enhancing data security. Strantzalis et al. [27] designed an edge AI system and developed various CNN models, which, after quantization and compression, were deployed on microcontroller units (MCUs) for real-time validation, achieving the detection of different sound signals. Hou et al. [28] introduced an innovative, compact, and low-power intelligent microsystem that leverages a CNN algorithm for sound-event recognition. With an average accuracy of over 92.50%, this system is well-suited for applications in home IoT and acoustic environment monitoring, particularly for enhancing safety. Yuh and Kang [29] developed a real-time sound monitoring system targeting elderly home care using a 2D CNN classifier; they deployed the system in real-world settings, achieving an accuracy of 95.55%. Chavdar et al. [30] employed a CNN to design a real-time remote acoustic monitoring system for assisted living scenarios, which was implemented on miniature MCUs and demonstrated effective detection performance.

This paper presents the design and implementation of an ASR system for elderly individuals that combines edge computing with deep learning technology. The system consists of edge devices equipped with multiple microphones, a wearable lanyard, and a small power supply, ensuring that the system is integrated into daily activities of the elderly and does not disrupt their routine. The edge devices are responsible for executing real-time audio processing and feature extraction to capture relevant acoustic features and performs real-time inference and recognition through algorithms. We developed four different deep learning models (CNN, long short-term memory (LSTM), bidirectional LSTM (BiLSTM), and deep neural network (DNN)) to analyze various acoustic features, enabling high-precision and real-time ASR. To optimize the deployment of models on edge devices, model quantization techniques were employed to effectively reduce computational complexity and memory usage. Additionally, the system is suitable for everyday wear, enhancing portability, and by processing data locally, it maximizes energy efficiency, reduces latency, and improves data security.

The contributions of this study are as follows:

(1) By processing data locally at the edge and performing real-time audio analysis and recognition, this approach enhances system responsiveness while also strengthening security and privacy, which is crucial for sensitive applications such as elderly care.
(2) We developed four deep learning models and applied model quantization techniques to reduce their complexity and memory usage, making them more efficient for deployment.
(3) The wearable design, featuring lightweight components, ensures ease of use of the system for the elderly, enabling its seamless integration into their daily activities while continuously providing ASR.

The remainder of the paper is organized as follows: Section 2 reviews related work. Section 3 presents the overall system architecture and foundational design of the system components. Section 4 discusses the data collection and preprocessing. Section 5 and Section 6 present the model construction methods and an analysis of the results, respectively. Finally, Section 7 summarizes our research findings and outlines future directions.

## 2. Related Work

Edge computing technology shifts data processing capabilities closer to the data source, thereby reducing the load of data transmission and enhancing real-time processing capabilities. This distributed computing architecture minimizes communication with the cloud and effectively addresses latency issues. Islam et al. [31] deployed an LSTM model on edge devices to build an ECI-TeleCaring system that enables real-time activity prediction and location awareness, thereby improving the quality of elderly care. Huang et al. [32] arranged smart sensors in the daily living spaces of the elderly; the system can recognize activities such as cooking, coughing, snoring, talking, listening to music, and walking, thereby offering benefits for home care. Gupta et al. [33] proposed a predictive model that combines edge computing and a CNN to develop an IoT-based health model. This system can provide timely health assistance to doctors and patients with an accuracy rate of 99.23%. Lian et al. [34] used inaudible acoustic sensing through home audio devices to detect fall incidents, analyzing actions via Doppler shifts and extracting features for classification. After dimensionality reduction and clustering, a hidden Markov model was used for training. Their system exhibited high performance and environmental adaptability.

ASR plays a pivotal role in monitoring systems. Advancements in deep learning have enhanced the detection of key sound events in daily life, thereby providing more efficient and reliable solutions for elderly care. For example, Shin [35] adapted the pre-trained YAMNet model for sound event detection in home care environments and developed a Y-MCC method based on the Matthews correlation coefficient (MCC) to generate new class mappings and improve event classification. Their experimental results demonstrated the excellent performance of the Y-MCC system across multiple datasets, particularly in SINS, ESC-50, and TUT-SED 2016. Kim et al. [36] developed a deep learning-based sound recognition model for effectively distinguishing emergency sounds in single-person households, thereby effectively detecting emergencies and enhancing personal safety and well-being. Pandya and Ghayvat [37] proposed an LSTM-CNN-based environmental acoustic event detection and classification system that achieved a classification accuracy of 77% under various noise conditions using a customized dataset.

Li et al. [38] developed an integrated learning auditory health monitoring system that detects everyday sounds and emergency events, such as falls, for elderly individuals at home, achieving an accuracy rate of 94.17% while also reducing privacy intrusion and enhancing monitoring efficiency. Ghayvat and Pandya [39] proposed an acoustic system designed to detect and recognize specific acoustic events in daily life. This system can process background sounds related to daily activities, enabling preventive health monitoring and incorporating audio signal processing and deep learning algorithms.

Although the real-time processing of large audio data on local devices reduces the latency associated with data transmission and significantly enhances responsiveness and reliability, the complexity of deep learning models often results in high computational and storage costs. This makes the direct deployment of resource-constrained edge devices challenging. Researchers have begun to adopt model quantization techniques, such as 8-bit post-training quantization (PTQ) and quantization-aware training (QAT), that reduce the computational complexity of models, thereby reducing their memory requirements and storage footprints [40]. Varam et al. [41] achieved a reduction in model size while maintaining good performance through float-16 quantization (F16) and QAT. Moreover, quantization techniques significantly improve energy efficiency, particularly under the low-power requirements of edge devices.

## 3. System Architecture

### 3.1. System Overview

**Figure 1:**
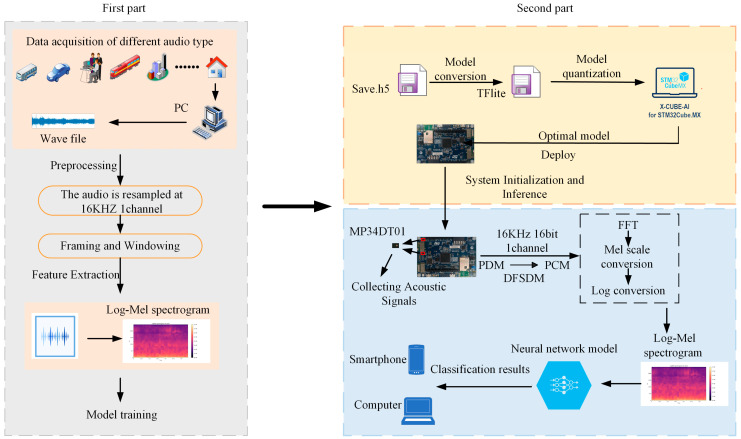
System architecture and functional overview.

This paper presents the development of an ASR system that integrates deep learning with low-power edge devices, tailored specifically for elderly monitoring. The low-power, wearable, and highly privacy-protective system is capable of real-time operation, as shown in Figure 1. The overall workflow comprises two main stages. First, audio data are processed on a PC, where raw audio samples are resampled to 16 kHz, segmented into frames, and transformed from the time domain to the frequency domain using a fast Fourier transform (FFT). Subsequently, the data are converted to the Mel scale using 30 overlapping triangular filters, producing 30 × 32 log-Mel spectrograms for subsequent deep learning model training.

In the second part, the first step involved saving the trained model in the .h5 file format. Subsequently, the model was converted into a TensorFlow Lite model for optimization. By utilizing integer-quantization techniques, the size of the model and computational complexity were reduced, thereby enhancing the inference efficiency of resource-constrained devices. The optimized model was deployed on the STM32L4 IoT Node Discovery Kit (STMicroelectronics, Geneva, Switzerland) using the STM32 Cube MX and Cube IDE, leveraging its built-in digital microphone for audio capture. The audio data were acquired in pulse density modulation (PDM) format and converted to a pulse code modulation (PCM) format using the digital filter for the sigma-delta modulation module. The PCM signal was segmented into rolling windows of 1024 samples, with Direct Memory Access (DMA) was used to receive the samples every millisecond. After receiving 512 PCM samples, FFT transformation and Mel filter application were performed to generate 32 columns of Mel-scaled spectrograms, which were then log-transformed to create input feature data for the neural network, and the final inference results were displayed on various devices.

### 3.2. Edge Computing Unit

The edge computing device used in this study is the STM32L4 IoT Node Discovery Kit, which was responsible for audio data acquisition and processing, and served as the deployment platform for deep learning model inference and recognition tasks. The technical specifications are presented in Table 1. This kit features an ultra-low-power STM32L475VGT6 MCU, equipped with 1 MB of flash memory and 128 KB of SRAM, and is powered by a 3.5 V DC supply. It integrates Bluetooth 4.1, Wi-Fi, NFC, and digital microphone modules, enabling a wide range of IoT applications with data collection across different communication protocols. It is particularly suitable for applications in ASM, smart home systems, and industrial automation. Its low-power design ensures long-term operation, making it ideal for continuous data collection while conserving energy.

**Figure 2:**
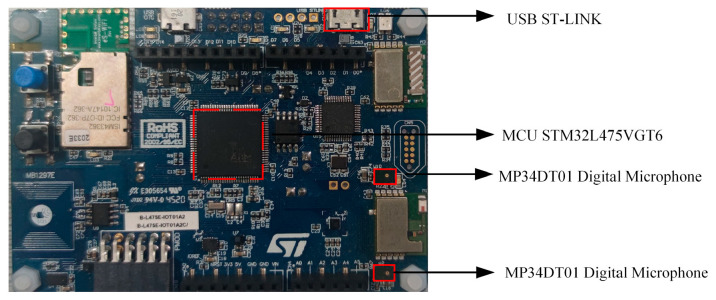
Schematic diagram of the STM32L4 IoT Node Discovery Kit.

Additionally, the audio capture device used in this study is integrated into the STM32L4 IoT Node Discovery Kit and comprises two ultra-compact, low-power ST-MEMS microphones (MP34DT01), as shown in Figure 2. These microphones utilize capacitive sensing elements and IC interfaces, with sensing elements manufactured through silicon micromachining specifically designed for audio sensors, allowing them to detect sound waves. The MP34DT01 microphones have an acoustic overload point of 120 dBSPL, a signal-to-noise ratio of 63 dB, and sensitivity of −26 dBFS. The microphones are spaced 21 mm apart to support beamforming algorithms, and they output digital signals in PDM format. A schematic diagram of the STM32L4 IoT Node Discovery Kit is shown in Figure 2.

### 3.3. Hardware Assembly

The hardware system consists of an STM32L4 IoT node as the core computing platform, equipped with the necessary sensors and processing capabilities for audio data collection and real-time analysis. It is attached to a wearable lanyard, allowing users to comfortably place the device on their chest, ensuring comfort and freedom of movement. The system is powered by an external power source that provides a consistent 3.5 V input to the STM32L4 board, enabling long-term continuous operation. This configuration ensures the high portability of the system, allowing it to autonomously perform ASR in real-world environments.

## 4. Data Collection and Processing

### 4.1. Data Acquisition

**Figure 3:**
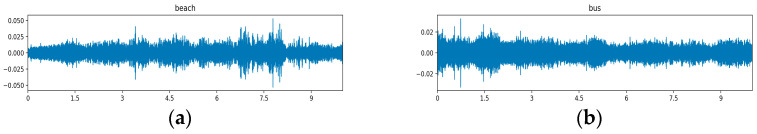
Sample audio waveforms of different acoustic scenes. (**a**) Beach waveform; (**b**) bus waveform.

To analyze and classify various environmental sound scenes, the datasets utilized in this experiment include the TUT Acoustic Scenes 2017 [42] and TAU Urban Acoustic Scenes 2020 Mobile Development datasets [43]. Both datasets were collected and constructed by the Tampere University of Technology, and encompass a variety of environmental audio scenes to ensure the diversity and representativeness of the experiment. The first dataset consists of audio recordings with each segment lasting 3–5 min, captured with a sampling rate of 44.1 kHz and a bit depth of 24 bits using binaural Soundman OKM II Klassik/studio A3 (Soundman, Berlin, Germany) electret in-ear microphones and a Roland Edirol R-09 (Roland Corporation, Hamamatsu, Japan) waveform recorder. Similarly, the second dataset features audio recordings with each segment lasting 2–3 min, captured at a sampling rate of 48 kHz and bit depth of 24 bits, using a combination of binaural Soundman OKM II Klassik/studio A3 electret in-ear microphones, a Zoom F8 recorder, and mobile phones. The schematic diagrams of different audio waveforms are shown in Figure 3.

In the selected datasets, we identified 17 common everyday life scenes, including bus, cafe/restaurant, car, city_center, forest_path, grocery_store, home, beach, library, metro_station, office with multiple people, residential_area, train, tram, urban park, shopping mall, and street pedestrian. Each scene category contained 312 audio recordings, which were trimmed into 10 s segments. All audio files were saved in the WAV format to facilitate subsequent analysis and processing. In addition, to ensure sample consistency and compatibility with the system processing capabilities, we resampled the audio data to 16 kHz and converted them into a single channel.

### 4.2. Audio Signal Preprocessing

Before training the neural network models, it is crucial to convert the audio signals into image signals. There are two prevalent methods for this conversion: log-Mel spectrogram and MFCC. The log-Mel spectrogram method directly converts an audio signal into a time-frequency representation, capturing essential audio features. In contrast, the MFCC method requires an additional mathematical transformation from the log-Mel spectrogram, which can lead to a significant loss of audio detail and increased computational overhead.

**Figure 4:**
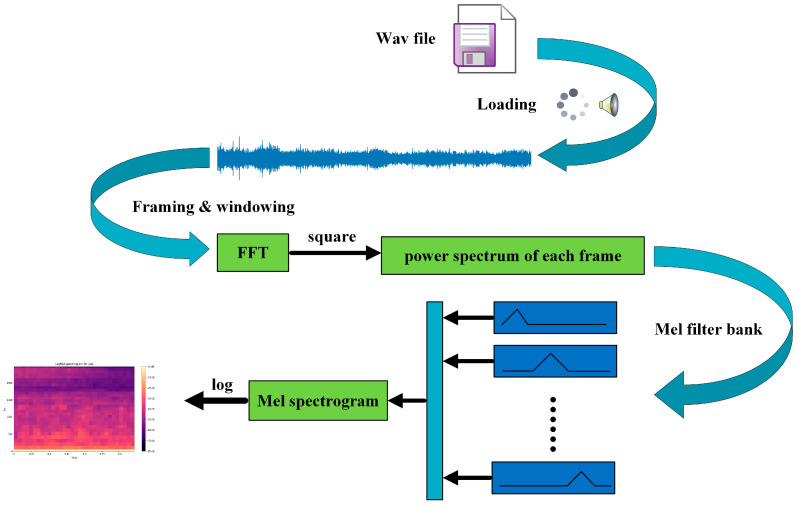
Feature extraction workflow diagram.

Given these considerations, this study used the log-Mel spectrogram for feature extraction. This method simplifies the process, reduces computational demands, and lowers energy consumption, all of which are essential for efficient neural network training. In addition, it preserves more of the original audio information, thereby enhancing the accuracy and responsiveness of the system. Figure 4 shows the feature extraction process using the log-Mel spectrogram method.

As shown in Figure 4, the audio wave files were first loaded at a sampling rate of 16 kHz and in mono to extract the time domain information of the audio signal. This step ensures that the data are standardized for subsequent processing. Next, the signal was segmented into frames, each with a length of 16,896 samples and frame interval (hop length) of 512 samples, and each frame windowed for short-time analysis. A Hanning window was used for the windowing process. The Hanning window was selected because of its ability to reduce spectral leakage by smoothly tapering the edges of each frame, thus minimizing abrupt changes that could introduce distortions in the frequency domain. The Hanning window formula is given by

(1) $$W\left(n\right)=\frac{1}{2}\left(1-\cos\left(\frac{2\pi n}{N-1}\right)\right)=\sin^{2}\frac{\pi n}{N-1},$$

where N is the number of samples per signal frame, and n=1,2,3…,n−1.

Subsequently, FFT is applied to each frame, converting the time domain signal into its frequency-domain representation; the FFT formula is given in (2). The FFT algorithm efficiently computes the discrete Fourier transform (DFT) by breaking the signal into sinusoidal components with varying frequencies and amplitudes, thereby decomposing the signal into its constituent frequencies and providing a spectrum for each frame.

(2) $$X\left(k\right)=\sum_{n=0}^{N-1}x\left(n\right)e^{\frac{-j2\pi nk}{N}},\;\left(0\leq K\leq N\right),$$

where $X\left(k\right)$ represents the spectrogram derived from the sound signals, $x\left(n\right)$ denotes the windowed signal, and N denotes the number of iterations involved in the Fourier transform sampling process.

Subsequently, the power spectrum is obtained by squaring the magnitude of the Fourier transform, which provides a measure of the power distribution of the signal across different frequencies. The power spectrum is given by

(3) $$P\left(k\right)=\frac{1}{N}{\left|X\left(k\right)\right|}^{2},$$

**Figure 5:**
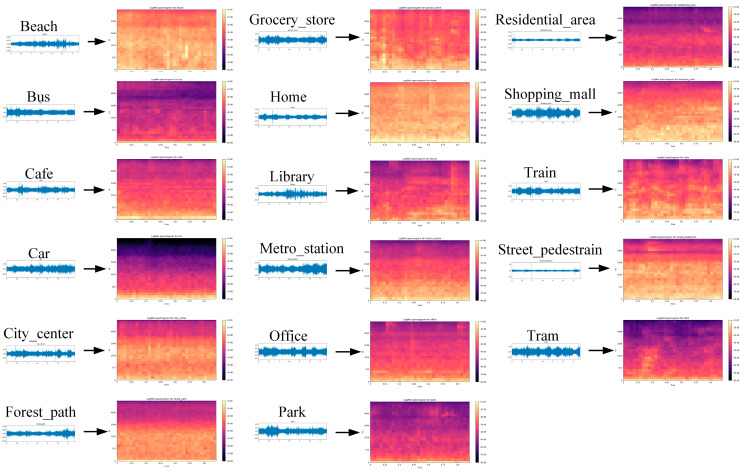
Different audio corresponds to feature extraction results.

Next, we selected a set of Mel triangular filter banks with equal heights, setting the number of filters to 30. The frequency response of the triangular filter $H_{m}\left(k\right)$ is calculated by (4). The power spectrum of each frame is multiplied by the Mel filter and summed in the time domain to obtain the Mel spectrogram, as shown in (5). Finally, the power spectrum is converted into decibel (dB) units to obtain the log-Mel spectrogram. All sounds undergo the aforementioned steps to produce log-Mel spectrograms, as shown in Figure 5.

(4) $$H_{m}\left(k\right)=\left\{\begin{array}{cc} 0 & k<f\left(m-1\right)\\ \frac{k-f\left(m-1\right)}{f\left(m\right)-f\left(m-1\right)} & f\left(m-1\right)\ll k\ll f\left(m\right)\\ \frac{f\left(m+1\right)-k}{f\left(m+1\right)-f\left(m\right)} & f\left(m\right)\ll k\ll f\left(m+1\right)\\ 0 & k>f\left(m+1\right) \end{array}\right.,$$

(5) $$S_{m}=\sum_{k=f\left(m-1\right)}^{f\left(m+1\right)}H_{m}\left(k\right)\cdot {\left|P\left(k\right)\right|}^{2},$$

where *m* represents the index of the filter, and $f\left(m-1\right)$, $f\left(m\right)$, and $f\left(m+1\right)$ correspond to the starting, mid, and ending points of the *m*-th filter, respectively. $S_{m}$ represents the Mel spectrogram, $P\left(k\right)$ denotes the power spectrum, and $H_{m}\left(k\right)$ is the frequency response of the Mel filter.

## 5. Proposed Deep Learning Models

### 5.1. Convolutional Neural Network (CNN) Model

Convolutional neural networks (CNNs) are deep learning models inspired by the hierarchical structure of the animal visual system, particularly effective in pattern recognition and feature extraction. Compared to other neural network architectures, CNNs are particularly valued for their ability to automatically learn hierarchical features from raw data, with a relatively simple yet powerful architecture that reduces the number of parameters, helping to prevent overfitting and improve model efficiency [44,45].

In the domain of audio recognition, CNNs convert raw audio signals into time frequency representations, such as log-Mel spectrograms or Mel-frequency cepstral coefficients (MFCCs), which are then treated as visual inputs. This approach enables CNNs to leverage their strong feature extraction capabilities, which are more efficient than traditional methods or other types of neural networks, such as fully connected networks (DNNs). CNNs are particularly effective in identifying local patterns, like pitch, rhythm, and timbre, making them well-suited for tasks like speech recognition, acoustic scene classification, and music genre identification. In contrast to other deep learning models, which often rely on manual feature engineering, CNNs can learn these patterns directly from the data, reducing the need for pre-processing and significantly improving model performance.

In a CNN, the core operation of convolution helps capture local patterns within these “image-like” representations of audio data. For a given input x and filter w, a single convolution operation at position (i,j) is defined as

(6) $$y_{i,j}={\left(x\;*\;w\right)}_{i,j}=\sum_{m=1}^{M}\sum_{n=1}^{N}x_{i+m,j+n}\cdot w_{m,n},$$

where x is the input feature map, w is the convolution kernel, $y_{i,j}$ is the value of the output feature map at position (i,j), and M,N represent the height and width of the convolution kernel, respectively. i,j denote the starting position indices of the current convolution operation in the input feature map. m,n are the indices of the elements in the convolution kernel.

To introduce nonlinearity, the CNN should apply an activation function, such as the ReLU function, to the convolved outputs. The ReLU function is defined as follows:

(7) $$ReLU\left(X\right)=\max\left(0,x\right),$$

After convolution and activation, pooling layers are used to reduce the spatial dimensions and enhance the model efficiency, with max pooling being the most common. For a feature map f, the max pooling in the p×p region is defined as

(8) $$z_{i,j}=\max\left\{f_{i+k,j+l}|0\leq k<p,0\leq l<p\right\},$$

where $z_{i,j}$ is the output value at position i,j, representing the maximum value within the pooling window. k and l are the relative indices within the pooling window, used to traverse each element within the p×p region.

**Figure 6:**
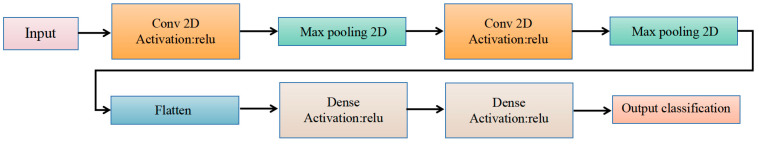
CNN model architecture.

In this study, we designed a multi-layer convolutional neural network (CNN) model for real-time acoustic scene recognition, specifically for elderly daily routines. The model begins with two convolutional layers, each with 32 filters of size 3×3 and ReLU activation functions. These layers extract intricate patterns from the raw acoustic signals. Following the convolutional layers, two 2×2 max-pooling layers are used to perform downsampling, reducing dimensionality and computational complexity while preserving key features. The resulting two-dimensional feature maps are flattened into a one-dimensional vector. The data are then processed through two fully connected layers: the first layer contains 32 neurons with ReLU activation, and the second has 17 neurons with softmax activation. This structure outputs a probability distribution over 17 classes, corresponding to various acoustic scene categories. The design aims to efficiently handle acoustic scene classification while being optimized for edge-based deployment. The structure of the constructed CNN model is shown in Figure 6.

### 5.2. Long Short-Term Memory (LSTM) Model

LSTM networks, a type of recurrent neural network (RNN) [46], extend traditional feedforward neural networks (FNNs) by incorporating recurrent connections that enable the model to capture sequential dependencies over time. Unlike standard RNNs, which struggle with long-term dependencies due to vanishing or exploding gradients, LSTMs are designed with specialized memory cells. These cells use three gates—forget, input, and output—to selectively retain, update, or discard information at each time step. This allows LSTMs to better manage long-range dependencies and retain important context over time, making them highly effective for tasks such as speech recognition, time series forecasting, and language modeling.

The forget gate determines which information from the previous cell state $C_{t-1}$ should be discarded. This is calculated as

(9) $$f_{t}=\sigma \left(W_{f}\cdot \left|h_{t-1},x_{t}\right|+b_{f}\right),$$

where $W_{f}$ and $b_{f}$ are the weight matrix and bias term, respectively, and $h_{t-1}$ and $x_{t}$ represent the hidden state from the previous time step and current input, respectively. σ is the Sigmoid activation function.

The input gate determines the new information to be added to the current cell state and is defined as

(10) $$i_{t}=\sigma \left(W_{i}\cdot \left|h_{t-1},x_{t}\right|+b_{i}\right),$$

Simultaneously, a candidate cell state ${\hat{C}}_{t}$ is generated to represent the new information to be potentially integrated as follows:

(11) $${\hat{C}}_{t}=\tanh\left(W_{C}\cdot \left|h_{t-1},x_{t}\right|+b_{C}\right),$$

where $W_{i}$, $W_{C}$ and $b_{i}$, $b_{C}$ are the weight matrices and bias terms, respectively, and tanh is the hyperbolic tangent activation function, ensuring that the values remain bounded.

The cell state is then updated by combining the information retained from the previous cell state with the new candidate information, as dictated by the forget and input gates:

(12) $$C_{t}=f_{t}\;*\;C_{t-1}+i_{t}\;*\;{\hat{C}}_{t},$$

Finally, the output gate controls the hidden state $h_{t}$, which is the LSTM output at time step *t*.

The output gate determines the parts of the cell state that contribute to the current hidden state $h_{t}$, defined as

(13) $$o_{t}=\sigma \left(W_{o}\cdot \left|h_{t-1},x_{t}\right|+b_{o}\right),$$

The final hidden state is then computed as

(14) $$h_{t}=o_{t}\;*\;\tanh\left(C_{t}\right),$$

where $h_{t}$ represents the output of the LSTM at time step h, where $W_{o}$ and $b_{o}$ are the weight matrix and bias term, respectively.

**Figure 7:**
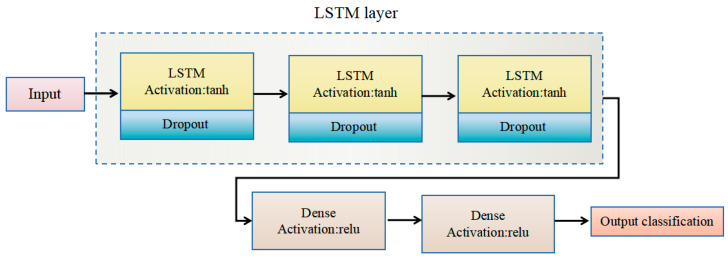
LSTM model architecture.

The gating mechanisms in LSTM networks enable them to capture time-dependent patterns and contextual information, making them ideal for tasks such as ASR. To leverage these capabilities of LSTM networks to improve the accuracy and reliability of audio scene recognition systems [47], this study constructed an LSTM model comprising three LSTM layers and two fully-connected layers. The input layer is an LSTM layer with 80 neurons, processing input sequences of length 30, with each time step containing 32 features. This is followed by two additional LSTM layers with 40 and 20 neurons, respectively. The fully-connected layers include a ReLU activation layer with 20 neurons and a softmax activation output layer with 17 neurons. A dropout layer was employed to reduce the risk of overfitting. The structure of the constructed LSTM model is shown in Figure 7.

### 5.3. Bidirectional Long Short-Term Memory (BiLSTM) Model

Bidirectional long short-term memory (BiLSTM) networks are an advanced variant of traditional LSTM networks, distinguished by their bidirectional architecture, which processes input data in both forward and backward directions. Unlike conventional unidirectional LSTMs, which rely solely on past information to make predictions, BiLSTMs enhance learning by simultaneously integrating both past (left-to-right) and future (right-to-left) contexts within the input sequence. This bidirectional processing enables BiLSTM networks to capture more comprehensive information, as they take into account both the preceding and subsequent data points.

The ability to incorporate future context, in addition to the past context, is especially beneficial for tasks that require a more complete understanding of the data, such as acoustic scene recognition and speech processing. In automatic speech recognition (ASR), for instance, this bidirectional approach allows BiLSTMs to better differentiate between temporally close or overlapping audio events, enhancing the model’s ability to distinguish subtle distinctions in sound. This makes BiLSTM networks particularly effective in real-world scenarios, where understanding the full context of a sound is crucial for accurate recognition [48].

BiLSTM networks process input sequences by leveraging both past and future context. For an input sequence $X=\left\{X_{1},X_{2},\ldots X_{T}\right\}$, the forward LSTM layer generates a hidden state ${\vec{h}}_{t}$ at each time step t by processing from $X_{1}$ to $X_{T}$ as follows:

(15) $${\vec{h}}_{t}={LSTM}_{forward}\left(x_{t},{\vec{h}}_{t-1}\right),$$

In parallel, the backward LSTM layer processes the sequence from $X_{T}$ to $X_{1}$, producing the hidden state, ${\overset{\leftarrow }{h}}_{t}$:

(16) $${\overset{\leftarrow }{h}}_{t}={LSTM}_{backward}\left(x_{t},{\overset{\leftarrow }{h}}_{t+1}\right),$$

The final hidden state at each time step t in the BiLSTM is obtained by concatenating the forward and backward hidden states as follows:

(17) $$h_{t}=\left|{\vec{h}}_{t};{\overset{\leftarrow }{h}}_{t}\right|,$$

**Figure 8:**
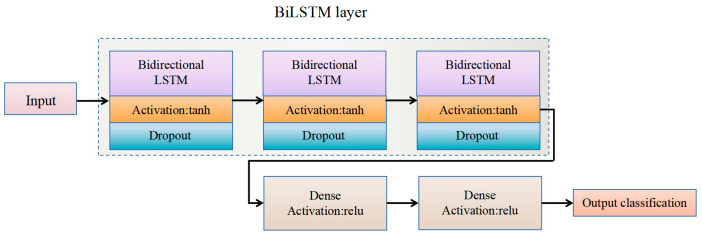
BiLSTM model architecture.

Similar to the previous model construction, the model built in this study comprises three bidirectional LSTM layers, followed by two fully connected layers. The input layer is an LSTM layer with 50 neurons, and the input sequence has a length of 30, with each time step containing 32 features. Subsequently, there are two BiLSTM layers, each with 25 neurons. The fully connected layers include a ReLU activation layer with 25 neurons and softmax activation output layer with 17 neurons. Dropout layers were used to mitigate the risk of overfitting. The structure of the constructed BiLSTM model is shown in Figure 8.

### 5.4. Deep Neural Network (DNN) Model

Deep neural networks (DNNs) are known for their ability to capture subtle features of various acoustic scenes, significantly enhancing the accuracy of recognizing and classifying different acoustic environments. Unlike traditional models, DNNs utilize multiple layers of interconnected nodes, enabling them to automatically learn intricate hierarchical representations of input data. This capacity to model complex relationships allows DNNs to detect fine-grained patterns in audio data, such as variations in frequency, duration, and context-specific acoustic cues.

The model architecture comprises an input layer, multiple hidden layers, and an output layer. In each hidden layer, numerous neurons perform a series of linear and nonlinear transformations on the input data, enabling the network to learn and progressively extract complex features and patterns [49]. For each neuron i in a hidden layer, the output $h_{i}^{\left(l\right)}$ at layer l is given by the following equation:

(18) $$h_{i}^{\left(l\right)}=\sigma \left(\sum_{j=1}^{n^{\left(l-1\right)}}w_{ij}^{\left(l\right)}h_{j}^{\left(l-1\right)}+b_{i}^{\left(l\right)}\right),$$

where $h_{j}^{\left(l-1\right)}$ is the output from the previous layer, $w_{ij}^{\left(l\right)}$ represents the weight between neurons j and i, and $b_{i}^{\left(l\right)}$ is the bias term. σ is the activation function.

**Figure 9:**
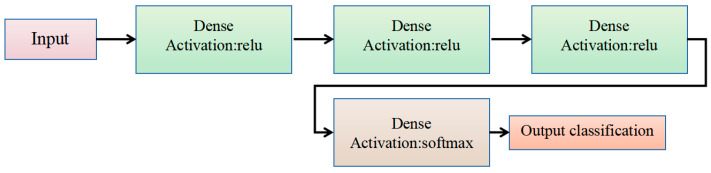
DNN model architecture.

The proposed DNN model comprises an input layer, followed by two hidden layers and an output layer. The input layer consists of a dense layer with 32 neurons using the ReLU activation function, accepting an input of a flattened feature vector with a length of 960. The two hidden layers each contain 32 neurons and also utilize the ReLU activation function, which aids in the learning of more abstract feature representations. The output layer employs the softmax activation function to classify the final task results. The structure of the constructed DNN model is shown in Figure 9.

## 6. Experimental Results and Analysis

### 6.1. Training Setup

This section discusses the training results of the four models. All trained models are based on the categorical cross-entropy loss function suitable for multi-class classification problems, with a learning rate of 0.01, and trained for 200 epochs using the SGD optimizer algorithm. The operating system used to train the models was Windows 10, running on an Intel(R) Core (TM) i9-10900 KF CPU @ 3.7 GHz with 64 GB of RAM. The graphics card used was an NVIDIA GeForce RTX 3080 (China, Yunnan, Kunming) with 10 GB of RAM. Python 3.8 was used as the programming language, and PyCharm was used as the development environment. The designed models were trained on the same dataset, with a training: validation: test set split of 7:2:1 to ensure fairness during the training process [50].

### 6.2. Model Evaluation

In this study, various evaluation metrics were used to assess the performance of the model, including accuracy, precision, recall, *F*1-*Score*, number of model parameters, and inference time.

Accuracy: Accuracy refers to the ratio of correctly predicted instances (both true positives and true negatives) to the total number of instances. This provides an overall measure of the model performance across all classes. Accuracy is calculated as follows:

(19) $$Accuracy=\frac{TP+TN}{TP+TN+FP+FN},$$

*Precision*: *Precision* is the ratio of correctly predicted positive instances (true positives) to all predicted positive instances. It is calculated as follows:

(20) $$Precision=\frac{TP}{TP+FP},$$

*Recall*: *Recall* is the ratio of the correctly predicted positive instances (true positives) to all actual positive instances in the dataset. It is calculated as follows:

(21) $$Recall=\frac{TP}{TP+FN},$$

*F*1-*Score*: *F*1-*Score* is the harmonic mean of precision and recall and is used to evaluate the overall performance of the model. It is calculated as follows:

(22) $$F1-Score=\frac{Precision\times Recall}{Precision+Recall}\times 2,$$

In the above formulas, TP and FP represent the numbers of true positive and false positive predictions, respectively, and TN and FN represent the numbers of true negative and false negative predictions, respectively.

### 6.3. Cross-Validation Method

During the model training process, we initially employed the traditional single-data split method [51]. Although this approach allows for quick model evaluation, it is susceptible to the influence of the data split and may not fully reflect the model’s stability and generalization ability under different data partitions. Specifically, a single-data split can lead to instability in the evaluation results, as different splits of the data may yield significantly different training and validation outcomes. To address this issue, we adopted the fivefold cross-validation method [52,53,54,55]. This technique divides the dataset into five folds, using four folds for training and the remaining fold as the validation set, repeating this process five times. This way, each data point serves as a validation set at least once, ensuring that every sample contributes to the model training and mitigating the potential bias caused by a single data split.

### 6.4. Result Analysis

Multiple evaluation metrics were used to compare the performance differences among the four models constructed in this study. The detailed results are presented in Table 2. Specifically, the CNN model achieved an accuracy of 98.5%, precision of 98.3%, *F*1-*Score* of 97.6%, and recall of 96.5%. The accuracy of the CNN was 8.7, 0.3, and 6.4 percentage points higher than those of the LSTM, BiLSTM, and DNN, respectively. Similarly, in terms of precision, the CNN outperformed LSTM, BiLSTM, and DNN by 9.2, 0.3, and 7.1 percentage points, respectively. The CNN also demonstrated superior recall and *F*1-*Score*, indicating its ability to effectively capture spatial hierarchy and local patterns in audio data, thereby achieving high classification performance and efficiency.

In terms of edge deployment, the model parameters and inference speed are critical factors. The CNN model had 31.3k parameters with an inference speed of 2.4 ms. While LSTM achieved a comparable inference speed, its parameter count was higher than that of CNN. Both BiLSTM and DNN demonstrated inferior performance in terms of computational resource usage and inference time compared to the CNN. In summary, the CNN emerged as the most effective model owing to its exceptional balance between high performance metrics and computational efficiency.

**Figure 10:**
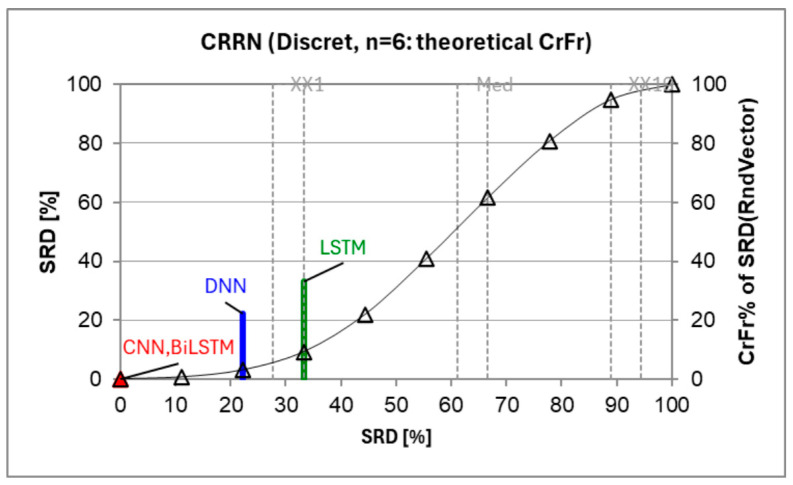
SRD result analysis.

Additionally, in Figure 10, the performance trends further emphasize CNN’s superiority in terms of stability and accuracy compared to the other models. The sum of ranking differences [56] (SRD) analysis results highlight that CNN slightly outperformed BiLSTM, as both models showed indistinguishable SRD values, but CNN demonstrated a marginally better consistency and stability. DNN, despite having a similar formula to CNN, performed worse, with a higher SRD indicating less stability. LSTM, with an SRD close to random ranking, performed the worst, showing significant inconsistency. Therefore, while both CNN and BiLSTM are reliable, CNN stands out as the better model overall.

**Figure 11:**
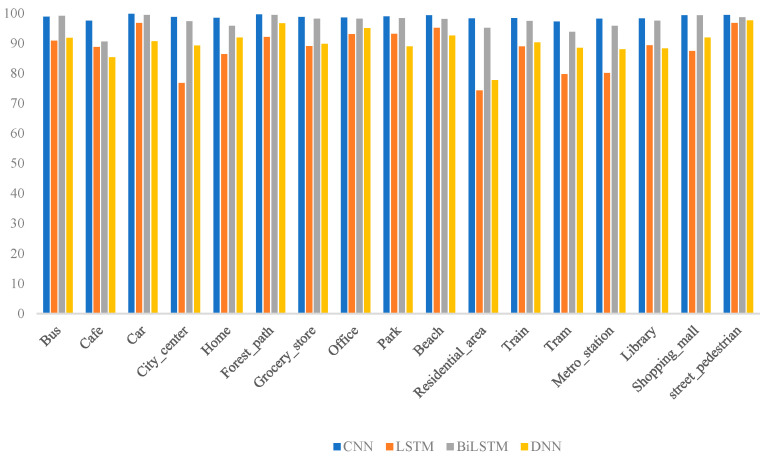
Recognition accuracy of different models in various acoustic scenes.

The recognition accuracies of the different deep learning models across various acoustic environments are presented in Figure 11. It is evident that the CNN and BiLSTM models performed similarly overall, with the CNN exhibiting superior accuracy in most scenes and achieving particularly high precision in specific environments. In contrast, the detection accuracies of the LSTM and DNN models were comparatively lower, particularly in complex noise scenes, such as City_center and Residential_area. This indicates that the CNN may be more suitable for deployment in dynamic soundscapes, where it can achieve a higher recognition accuracy.

**Figure 12:**
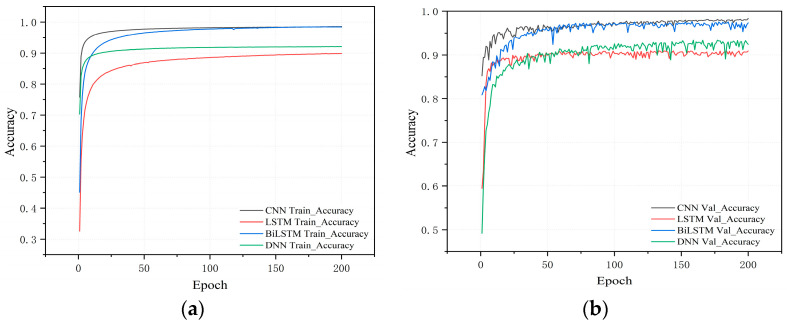

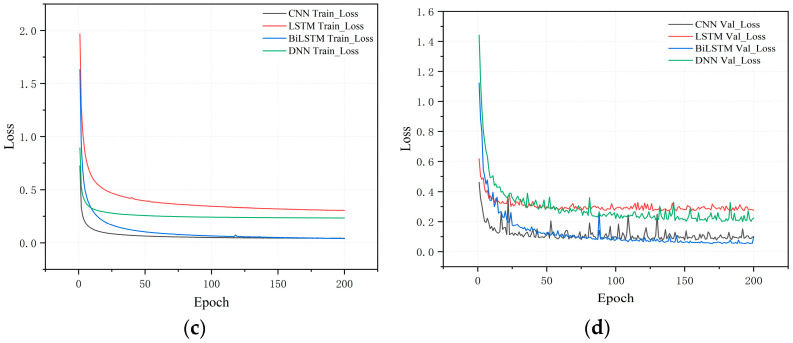
Accuracy and loss progression of different models. (**a**,**b**) The training and validation accuracy progression, respectively; (**c**,**d**) the training and validation loss progression, respectively.

The trajectories of the training and validation accuracy and loss for the four deep learning models are shown in Figure 12. All models exhibited a significant upward trend in accuracy during the initial training stages, with the accuracy curve of the CNN model rising sharply and quickly converging to its maximum achievable accuracy. This indicates that the CNN model is highly capable of effectively learning relevant features from the training data. In contrast, the accuracy improvement for the LSTM and DNN models was more gradual, whereas the BiLSTM model, although closer to the CNN in terms of performance, demonstrated lower efficiency. Regarding the loss curves, the DNN and LSTM models converged more slowly, with relatively higher loss values. Although the final loss values of the CNN and BiLSTM models were similar, the CNN demonstrated superior efficiency. Overall, the CNN model stands out for its stable and rapid convergence, making it the most effective architecture for reducing loss and minimizing prediction error compared with the other three models.

**Figure 13:**
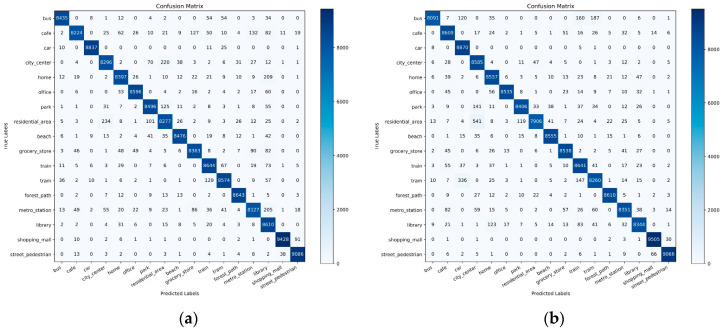

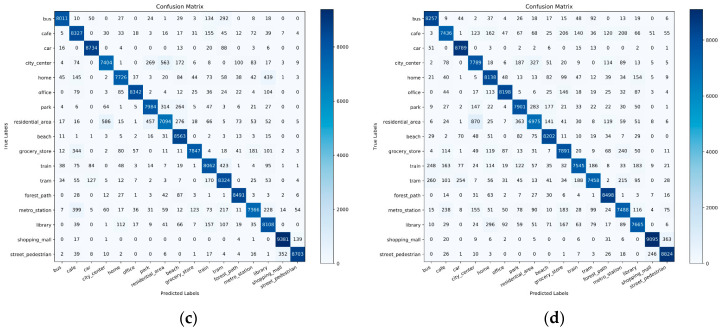
Confusion matrices generated by different models. (**a**) Confusion matrix of the CNN model; (**b**) confusion matrix of the BiLSTM model; (**c**) confusion matrix of the LSTM model; (**d**) confusion matrix of the DNN model.

We constructed confusion matrices for each of the four models to further analyze their classification performance in ASR tasks related to the daily activities of the elderly, as shown in Figure 13. First, from the CNN confusion matrix, it is evident that this model demonstrated highly accurate classification across nearly all categories, with particularly low misclassification rates for frequently occurring scene categories. However, minor confusion remained between similar scenes, such as “home” and “library.” The LSTM and DNN models, which have a relatively lower accuracy, showed a higher number of misclassifications in complex or acoustically similar scenes, as observed from their confusion matrices, indicating their limited ability to capture intricate audio features. The performance of the BiLSTM model fell between those of the CNN and LSTM; while its accuracy approached that of the CNN, it still showed some degree of misclassification in challenging scenes, such as City_center and Residential_area. Overall, the confusion matrix of the CNN model showed the best performance with the lowest misclassification rate, effectively identifying all acoustic scenes.

In Table 3, we employed the cross-validation method for model evaluation. The results show that the CNN outperformed all other models, achieving the highest accuracy of 98.51% and the lowest standard deviation of 0.03, demonstrating exceptional stability and reliability. The performance of CNN was almost unaffected by data partitioning, which fully validates its stability across different data splits. Compared to other models, CNN significantly outperformed BiLSTM in both accuracy and consistency. Although BiLSTM’s performance was also close, with an accuracy of 98.19%, its stability was slightly lower, and its performance showed greater fluctuations. Both LSTM and DNN exhibited lower accuracy and larger standard deviations, indicating poorer stability and overall performance compared to CNN and BiLSTM. Based on this analysis, CNN is considered the most suitable model for elderly acoustic scene recognition, especially in applications that require high accuracy and consistent performance.

Deploying a trained 32-bit floating-point model directly on edge devices is impractical due to resource constraints. Typically, quantization techniques must be applied to convert the model into an 8-bit fixed-point format. Two commonly used methods are PTQ and QAT. PTQ applies quantization after the model has been fully trained, thereby reducing storage and computation by converting the floating-point model into a lower-precision integer model. Although this method is straightforward and efficient, it can lead to a loss of accuracy. By contrast, QAT simulates the effects of quantization during the training process and optimizes the model weights via backpropagation to minimize quantization errors. Although QAT yields a higher post-quantization accuracy, it is more complex and time-consuming. In this study, we employed an 8-bit PTQ, and the resource usage results for different models after quantization are listed in Table 3.

The results in Table 4 indicate that compared to the other three models, the CNN model achieved the best balance in terms of Flash and RAM usage, computational cost, and training time, making it well-suited for deployment on resource-constrained edge devices. Although the performance of the LSTM model was close to that of the CNN, its memory usage and training cost were slightly higher. Despite its greater complexity, the BiLSTM model made excessive demands on memory and computational resources, and had the longest training time, which limited its applicability in real-time applications. Although the DNN model had the lowest computational cost and highest training speed; its high RAM usage may pose challenges for its deployment on devices with limited memory. Therefore, the CNN stands out as the most suitable choice among the four models for high-performance, low-resource edge computing scenes.

### 6.5. Evaluation of Real-World Performance

#### 6.5.1. Smartphone Application

**Figure 14:**
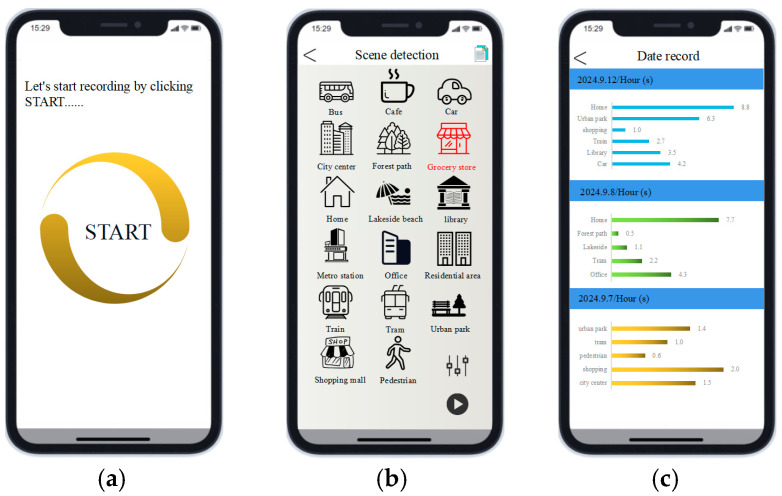
Smartphone application interface display: (**a**) start detection; (**b**) real-time detection results, and (**c**) data recording results.

We developed a user-friendly smartphone application specifically designed for elderly users, offering simple and intuitive operation. The application aims to provide convenient acoustic scene monitoring and health management. As shown in Figure 14, the interface features real-time acoustic scene monitoring, with the current scene highlighted in red. Various settings options allow users to adjust the volume, start or pause recordings according to their preferences, and check the duration spent in each scene. This helps users track their daily activities and make informed decisions accordingly to ensure safety and well-being.

#### 6.5.2. Different Scenario Settings and Test Results

**Figure 15:**
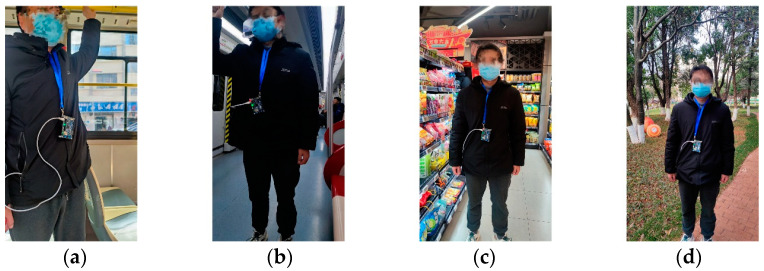
Examples of different acoustic scenes. (**a**) Bus scene; (**b**) tram scene; (**c**) shopping scene; (**d**) park scene.

To ensure the safety and effectiveness of the evaluation process, we selected 17 volunteers (8 males and 9 females) to simulate the daily activities of elderly users. This approach minimized potential safety risks and allowed for better control over the experimental environment. Multiple trial scenarios were created, as shown in Figure 15. During the evaluation, the volunteers, wearing the device, engaged in activities within designated areas, purposefully mimicking the natural movements and interactions typical of elderly users. This method provided a realistic representation of elderly behaviors, allowing us to assess the reliability, applicability, and overall effectiveness of the system in accurately monitoring a variety of acoustic scenes.

Table 5 presents the evaluation results across different scenes, which reveal that the system achieved an average accuracy rate exceeding 90% in the examined environments. Notably, the accuracy rates reached 93.3%, 95.4%, and 91.1% in the noisy environments of bus, tram, and shopping scenes, respectively. Conversely, in the relatively quiet setting of park scene, the detection accuracy decreased to 90.6%. This reduction can be attributed to the lower sound levels in quieter environments, which make it relatively challenging to capture and analyze performance outcomes. In addition, during the evaluation phase, users reported that the operation was user-friendly and did not interfere with their daily activities, indicating a positive user experience and high applicability. Overall, the system demonstrated high precision and stability across various acoustic scenarios, validating its reliability and practicality in real-world applications.

## 7. Discussion

### 7.1. Comparison with Other Methods

As shown in Table 6, our proposed CNN model achieved the highest accuracy (98.5%) among the compared approaches while maintaining a minimal memory footprint (6.5 KB). This makes it highly suitable for deployment on resource-constrained edge devices. Compared to Strantzalis et al. [27] (97.8% accuracy, 7.0 KB) and Hou et al. [28] (94.9% accuracy, 3.58 KB), our model not only outperformed in classification performance but also maintained efficiency in memory usage. While Lee et al. [12] achieved competitive accuracy (92.9%), its significantly larger model size (204.3 KB) limited its feasibility for edge deployment. Similarly, Ranmal et al. [57], employing an ESC-NAS approach, has a lower CV score (85.78%) and a substantially larger memory requirement (365 KB), making it less efficient for real-time applications. However, it is important to note that some alternative models, such as Lee et al.’s [58] (ResNet-based, 93.2% accuracy), leverage different architectures that may provide robustness to specific conditions at the cost of increased model size (31.3 KB). While our model achieved the best balance in terms of accuracy, efficiency, and real-time feasibility, future work will explore improving its robustness under challenging acoustic conditions without increasing computational complexity.

### 7.2. Comparison of Different Optimization Methods

In Table 7, we explored the trade-offs between accuracy and computational complexity for various optimization methods. PTQ provides a significant reduction in memory usage, dropping model size to just 47 KB. This is achieved while maintaining a high accuracy of 98.5%. In contrast, methods like pruning and hybrid-magnitude pruning also reduce model size, but they typically involve some loss in accuracy: pruning, for example, results in an accuracy of 82.3%, and hybrid-magnitude pruning achieves 85.6%. Knowledge distillation, on the other hand, retains accuracy (98.6%) while reducing model size to 194.5 KB, but still does not achieve the memory efficiency of PTQ.

## 8. Summary and Conclusions

In this work, we designed an acoustic scene recognition (ASR) system aimed at monitoring the daily activities of elderly individuals. By processing real-time audio signals, the system promptly identifies key acoustic features in the daily environment, enabling accurate and timely scene recognition. This approach addresses the critical need for effective real-time monitoring in elderly care. To enhance the system’s deployment on edge devices, we developed several deep learning models and employed model quantization techniques, significantly reducing computational complexity and memory consumption. Experimental results demonstrated that, among the models trained and tested on the same dataset, the CNN model outperformed others, achieving an accuracy of 98.5% while maintaining low computational requirements. This enables the system to provide high-precision real-time monitoring with minimal power consumption, thus improving the overall efficiency of elderly care systems. However, it is important to note that the performance advantages of the CNN model are specific to the dataset and experimental conditions used in this study and should not be generalized to all environments or applications.

Building on this foundation, the system’s stability was tested across different participants, with real-world evaluations confirming its robustness in reliably identifying acoustic environments across various scenarios. Furthermore, the user-friendly smartphone application effectively addresses the care needs of the elderly by providing practical, accessible real-time environmental monitoring and personalized health management.

However, several challenges remain unaddressed. In unpredictable settings, the sensitivity of the system to environmental noise can affect detection accuracy. Future work could focus on developing optimized, lightweight deep learning algorithms to enhance model accuracy in complex and noisy environments. Additionally, implementing an event-triggered mechanism can help reduce overall power consumption by ensuring that the system is activated only upon detecting specific events. Finally, ongoing data collection from diverse real-world acoustic environments will further expand the dataset, enhancing the system’s generalization capabilities across various soundscapes and improving its robustness and adaptability.

## Figures and Tables

**Table 1:** Technical specifications.

No.	Parameter	Specification
1	Operating temp	−40–+85 °C
2	Dimensions	90 mm × 60 mm
3	Microphone type	MP34DT01
4	Signal-to-noise ratio	63 dB
5	Sensitivity value	−26 dBFS
6	Voltage	3.5 V

**Table 2:** Comparison of different deep learning models.

Model	Accuracy (%)	*Precision* (%)	*F*1 (%)	*Recall* (%)	Parameter (k)	Inference Time (ms)
CNN	98.5	98.3	97.6	96.5	47.0	2.4
LSTM	89.8	89.1	88.4	90.0	61.7	5.5
BiLSTM	98.2	98.0	96.9	96.6	75.3	10.1
DNN	92.1	91.2	90.7	91.9	32.4	6.5

**Table 3:** Cross-validation results.

Model	1st-Fold	2nd-Fold	3rd-Fold	4th-Fold	5th-Fold	Average ± Std
CNN	98.47	98.51	98.48	98.55	98.52	98.51 ± 0.03
LSTM	89.11	89.68	89.32	89.81	89.70	89.52 ± 0.26
BiLSTM	98.03	98.35	98.29	98.14	98.12	98.19 ± 0.12
DNN	92.49	91.64	92.13	91.85	91.21	91.86 ± 0.43

**Table 4:** Comparison of different model parameters.

Model	Flash (KB)	Ram (KB)	MACC	Training Time (s)
CNN	25.6	6.5	509,179	21,356.72
LSTM	28.1	7.2	506,568	45,919.08
BiLSTM	22.4	9.4	682,364	74,526.05
DNN	33.2	16.3	33,635	1014.58

**Table 5:** Test results for different scenes.

Scene	Accuracy (%)
Bus	93.3
Tram	95.4
Shopping	91.1
Park	90.6

**Table 6:** Comparison of Different Model Methods.

Study	Method	Feature Extraction	Performance	Ram/Size (KB)	Real-Time
Lee, Y et al. [12]	CNN	Log-Mel spectrogram	Accuracy: 92.9%	204.3	No
Strantzalis, K et al. [27]	CNN	MFCC	Accuracy: 97.8%	7.0	Yes
Hou, L et al. [28]	CNN	MFSC	Accuracy: 94.9%	3.58	Yes
Ranmal, D et al. [57]	ESC-NAS	N/A	CV Score: 85.78%	365	Yes
Lee, C et al. [58]	ResNet	Mel-spectrogram	Accuracy: 93.2%	31.3	No
This work	CNN	Log-Mel spectrogram	Accuracy: 98.5%	6.5	Yes

**Table 7:** Comparison of Different Optimization Methods.

Study	Method	Accuracy	Impact on Memory Usage	Size (KB)
Mou, A and Milanova, M [59]	Pruning	82.3%	Reduces model size by eliminating unnecessary weights	528
Priebe, D et al. [60]	Knowledge Distillation	98.6%	Reduces model size, retains accuracy	194.5
Paranayapa, T et al. [61]	Hybrid-Magnitude Pruning	85.6%	Further reduces model size compared to standard pruning	122.8
This work	PTQ	98.5%	Significant reduction in memory usage	47

## Data Availability

Data are contained within the article.
